# Dual‐Targeting Oligopeptide‐215 Regulates Skin Barrier Homeostasis Through Concurrent Modulation of JAK–STAT and NF‐κB Signaling

**DOI:** 10.1111/jocd.70704

**Published:** 2026-03-19

**Authors:** Qianqian Zhang, Chuanyuan Hu, Yujing Chen, Yanrong Chen, Chaowan Guo, Zijian Liu, Wenfeng Ding

**Affiliations:** ^1^ Shenzhen Winkey Technology Co. Ltd. Shenzhen China; ^2^ Guangdong Marubi Biotechnology Co. Ltd. Guangzhou China

**Keywords:** barrier factors, inflammation regulation, JAK–STAT signaling, NF‐kB signaling, Oligopeptide‐215, skin barrier

## Abstract

**Objective:**

To evaluate Oligopeptide‐215, a biomimetic peptide engineered from amphibian skin secretions, for its dual capacity to restore skin barrier homeostasis by concurrently targeting physical barrier proteins and inflammatory pathways via JAK–STAT and NF‐κB modulation.

**Methods:**

In vitro models used UVB‐damaged HaCaT keratinocytes, LPS‐stimulated RAW264.7 macrophages, and IL‐4/IL‐13‐stimulated HaCaT cells. Assays included cell viability (MTT), migration (scratch), adhesion, barrier proteins (FLG/LOR; ELISA/IF), signaling phosphoproteins (pJAK1/pTYK2/pSTAT3/pSTAT6/pNF‐κB; IF), and inflammatory mediators (NO/PGE₂/TNF‐α/IL‐6/IL‐1β; ELISA).

**Results:**

Oligopeptide‐215 antagonizes IL‐13/IL‐4‐mediated JAK–STAT6/STAT3 signaling, thereby restoring expression of critical barrier proteins FLG and LOR. Additionally, it suppresses inflammatory responses by inhibiting NF‐κB‐dependent cytokine production. In UVB‐damaged keratinocytes, Oligopeptide‐215 promotes cellular repair through enhanced proliferation, adhesion, and migration.

**Conclusion:**

These findings revealed an innovative repairing mechanism combining physical barrier with immunologic barrier, and established Oligopeptide‐215 as a potential skincare ingredient for skin barrier dysfunction.

## 1 Introduction

The skin barrier represents the body's first line of defense against environmental insults, serving as both a physical and immunological interface [1]. Disruption of this barrier is increasingly recognized as a central factor in various dermatological conditions, from atopic dermatitis to aging‐related disorders [2, 3]. The functional integrity of the skin barrier depends on a complex interplay between structural proteins, lipid organization, and immune responses [4, 5]. Despite the clinical significance of barrier dysfunction, current therapeutic approaches often fail to address this multifaceted nature of barrier homeostasis, particularly the intricate relationship between structural integrity and inflammatory responses that characterize compromised barrier states [6, 7, 8].

Recent advances have highlighted the role of cytokine‐mediated signaling in barrier homeostasis, particularly the IL‐13/IL‐4 axis [9, 10]. These cytokines, through JAK–STAT pathway activation, can suppress the expression of critical barrier proteins such as filaggrin and loricrin [11, 12, 13]. The importance of this pathway is underscored by clinical observations showing elevated IL‐4/IL‐13 signaling in various skin disorders characterized by barrier dysfunction [14, 15, 16]. However, the molecular mechanisms linking inflammatory signaling to barrier protein regulation remain incompletely understood. This knowledge gap has hampered the development of targeted therapies that could simultaneously address both barrier dysfunction and associated inflammation [17, 18, 19].

The therapeutic potential of naturally occurring peptides in skin barrier protection has garnered increasing attention [20]. These peptides often exhibit multiple bioactive properties, including antimicrobial, anti‐inflammatory, and wound‐healing activities [21, 22]. However, their clinical application is frequently limited by poor stability, inadequate penetration, and incomplete mechanistic characterization. The challenge lies in developing peptide‐based therapeutics that maintain biological efficacy while overcoming these pharmaceutical limitations.

Amphibian skin secretions have emerged as a rich source of bioactive peptides with potential therapeutic applications [23, 24]. These peptides have evolved to function in both defensive and healing capacities, making them particularly relevant for human skin applications [25]. We previously identified CW49, a peptide from amphibian skin secretions with promising barrier‐protective properties [26]. However, like many naturally occurring peptides, its clinical utility was limited by oxidative instability.

Here, we designed a biomimetic peptide through strategic amino acid substitution. By replacing the oxidation‐susceptible cysteine residue in the parent peptide CW49 with methionine, we achieved Oligopeptide‐215, which showed exceptional thermodynamic stability while preserving biological activity. Our comprehensive results revealed that Oligopeptide‐215 antagonized IL‐13/IL‐4‐mediated suppression of barrier proteins through JAK–STAT pathway modulation while simultaneously regulating inflammatory responses. By regulating both physical barrier and immunological barriers, Oligopeptide‐215 represented a novel dual‐mechanism approach for repairing skin barrier dysfunction. This unique dual‐targeting capability represents a significant advance in peptide‐based barrier protection strategies. Furthermore, our findings demonstrate that biomimetic peptide can be developed into more effective skincare agents for repairing the skin barrier.

## 2 Materials and Methods

Oligopeptide‐215 was supplied by Shenzhen Winkey Technology Co. Ltd. (Shenzhen, China). Cell culture reagents, including Dulbecco's Modified Eagle Medium (DMEM) and fetal bovine serum (FBS), were sourced from Thermo Fisher Scientific (Carlsbad, CA, USA) and Clark Bioscience (Claymont, DE, USA), respectively. Antibiotics (penicillin and streptomycin) came from Biosharp (Hefei, China). MTT and DAPI were acquired from Sigma‐Aldrich (St. Louis, MO, USA). For protein analysis, RIPA lysis buffer and the BCA protein assay kit were obtained from Beyotime Biotechnology (Jiangsu, China). ELISA kits for detecting IL‐6, TNF‐α, IL‐1β, FLG, and LOR were procured from CUSABIO Biotechnology (Wuhan, China). Polyclonal primary antibodies against pJAK1 (I:100 for IF), pTYK2 (I:100–200 for IF), pSTAT3 (I:100 for IF) and pSTAT6 (I:100 for IF) were purchased from The Proteintech Group (Wuhan, China). Polyclonal primary antibodies against pNF‐kB and NO kits were purchased from Beyotime (Shanghai, China). Polyclonal primary antibodies against FLG and LOR were obtained from The Proteintech Group, and were diluted 1:100 for IF. The PGE_2_ ELISA kit is obtained from EIAab (Wuhan, China).

### 2.1 Synthesis and Characterization of Oligopeptide‐215

The peptide Ac‐APFRMGIMTTN‐amide was synthesized on a 0.1 mmol scale via standard Fmoc solid‐phase peptide synthesis (SPPS) using Rink amide‐AM resin. For each coupling cycle, a 4‐fold molar excess of Fmoc‐protected amino acids was activated with HATU/HOBT before being introduced to the resin. Upon sequence completion, the peptide was cleaved from the resin using a trifluoroacetic acid/thioanisole/anisole mixture (95:3:2, v/v). After 2.5 h, the product was precipitated in cold diethyl ether. Crude peptide purification was performed by reversed‐phase HPLC (RP‐HPLC) on a C18 column (4.6 × 250 mm) with a 0.1% TFA/acetonitrile gradient (5% to 20% over 30 min). Final product identity was confirmed by electrospray ionization mass spectrometry (ESI‐MS).

### 2.2 Cell Line and Culture Conditions

HaCaT human keratinocytes and RAW264.7 murine macrophages were acquired from the American Type Culture Collection (ATCC). Both cell lines were maintained in DMEM supplemented with 10% FBS and 1% penicillin–streptomycin, under standard culture conditions (37°C, 5% CO_2_, humidified atmosphere). For subculturing, HaCaT cells were detached using 0.25% trypsin, whereas RAW264.7 cells were passaged mechanically without trypsinization.

### 2.3 Clearance of NO Production

2 × 10^4^ cells/well were seeded in a 96‐well plate and incubated for 24 h. The culture medium was substituted by 100 μL of Oligopeptide‐215 solution at different concentrations and 1 μg/mL of LPS co‐incubated for 24 h, and 10 μg/mL of Dexamethasone (Dex) as a positive control. The content of NO in the supernatants was detected according to the manufacturer's protocol of the Total Nitric Oxide assay kit.

### 2.4 Cell Viability Assay

Cell proliferation was assessed via the MTT assay. Briefly, cells were seeded in 96‐well plates and incubated under standard conditions (37°C, 5% CO_2_) for 24 h to reach 30%–50% confluence. Subsequently, the medium was replaced with serum‐free medium containing varying concentrations of Oligopeptide‐215 (12.5, 25, 50, 100 ppm) for another 24 h. Following treatment, cells were washed twice with PBS and subjected to UVB irradiation (50 mJ/cm^2^) under a thin layer of PBS using a dedicated irradiator. Control cells were handled identically but without UVB exposure. After irradiation and an additional PBS wash, 10 μL of MTT solution (1 mg/mL) was added to each well containing 100 μL of fresh medium, followed by 2 h of incubation. The resulting formazan crystals were dissolved in DMSO, and absorbance was measured at 570 nm using a microplate reader. Three independent replicates were performed for each experiment.

### 2.5 In Vitro Scratch Assay

Cell migration was assessed in vitro via a scratch wound healing assay. Briefly, cells were seeded at high density into 6‐well plates and cultured in DMEM with 10% FBS until reaching 80%–90% confluence (approximately 48 h). A uniform wound was then generated in each monolayer using a p200 pipette tip. After three washes with PBS to remove detached cells, the cells were incubated for 12 h in Opti‐MEM medium containing Oligopeptide‐215 at the indicated concentrations (25, 50, and 100 ppm). Wound areas were photographed under a microscope immediately after scratching (0 h) and at the 12 h time point. The images were analyzed using Image‐Pro Plus software to quantify the remaining wound area. The relative migration rate was calculated by normalizing the wound area at 12 h to the initial (0 h) wound area.

### 2.6 Determination of FLG and LOR

HaCaT cells were seeded into a 96‐well plate with appropriate density, cultured for 24 h, irradiated with UVB (50 mJ/cm^2^) using a UVB irradiation machine. Then, cells were treated with different concentrations of Oligopeptide‐215 (12.5, 25, 50, 100 ppm) in serum‐free medium conditions for 24 h. The supernatant fractions were collected and immediately used for Elisa assay or stored at −20°C for later use. Then, cells were lysed on ice with RIPA lysate for 20 min, during which time the cells were blown repeatedly with a pipette gun for full lysate. Total protein concentration in whole cells was tested using the BCA kit. The contents of FLG and LOR were determined in strict accordance with the corresponding Elisa kit instructions.

In another assay, the HaCaT cells were stimulated with 100 ng/mL IL‐4/IL‐13 and co‐incubated with different concentrations of Oligopeptide‐215 for 24 h.

### 2.7 Immunofluorescence

Cells were washed twice with PBS, permeabilized in 0.1% Triton X‐100 overnight at 4°C. After the fixation procedure, the sections were cryoprotected in a PBS solution supplemented with 0.9 mol/L of sucrose overnight at 4°C. The primary antibodies used in the present study were as follows: pJAK1 (1:100), pSTAT3 (1:100), pTYK2 (1:100), and pSTAT6 (1:100). Subsequently, samples were incubated with secondary antibody (1:5000–1:10 000) for 1 h at room temperature. DAPI was used to label the nuclei, and images were captured using an inverted microscope.

### 2.8 Determination of IL‐6, IL‐8, IL‐1β, TNF‐α, and PEG_2_

The HaCaT cells or RAW264.7 cells were dispensed into 12‐well plates and permitted to proliferate for a duration of 24 h. Subsequently, the complete culture medium was aspirated from the wells, and PBS was introduced for a starvation treatment period of 3 h. For the blank control group, the wells were replenished with complete culture medium. The LPS group received LPS in conjunction with complete culture medium. Additionally, the sample groups were treated with LPS, complete culture medium, and diluted samples for a total of 48 h. Following the incubation period, the cells were harvested, centrifuged to separate the supernatant, and stored in a freezer maintained at −80°C. The IL‐6, IL‐8, IL‐1β, TNF‐α and PEG_2_ ELISA assays were conducted in accordance with the manufacturer's instructions.

### 2.9 Statistical Analysis

Data are expressed as the mean ± standard deviation (S.D.) of at least three independent experiments. Differences between groups were determined using one‐way analysis of variance and Student's *t*‐test. *p* < 0.05 was considered statistically significant.

## 3 Results

### 3.1 Oligopeptide‐215 Design and Characterization

**FIGURE 1:**
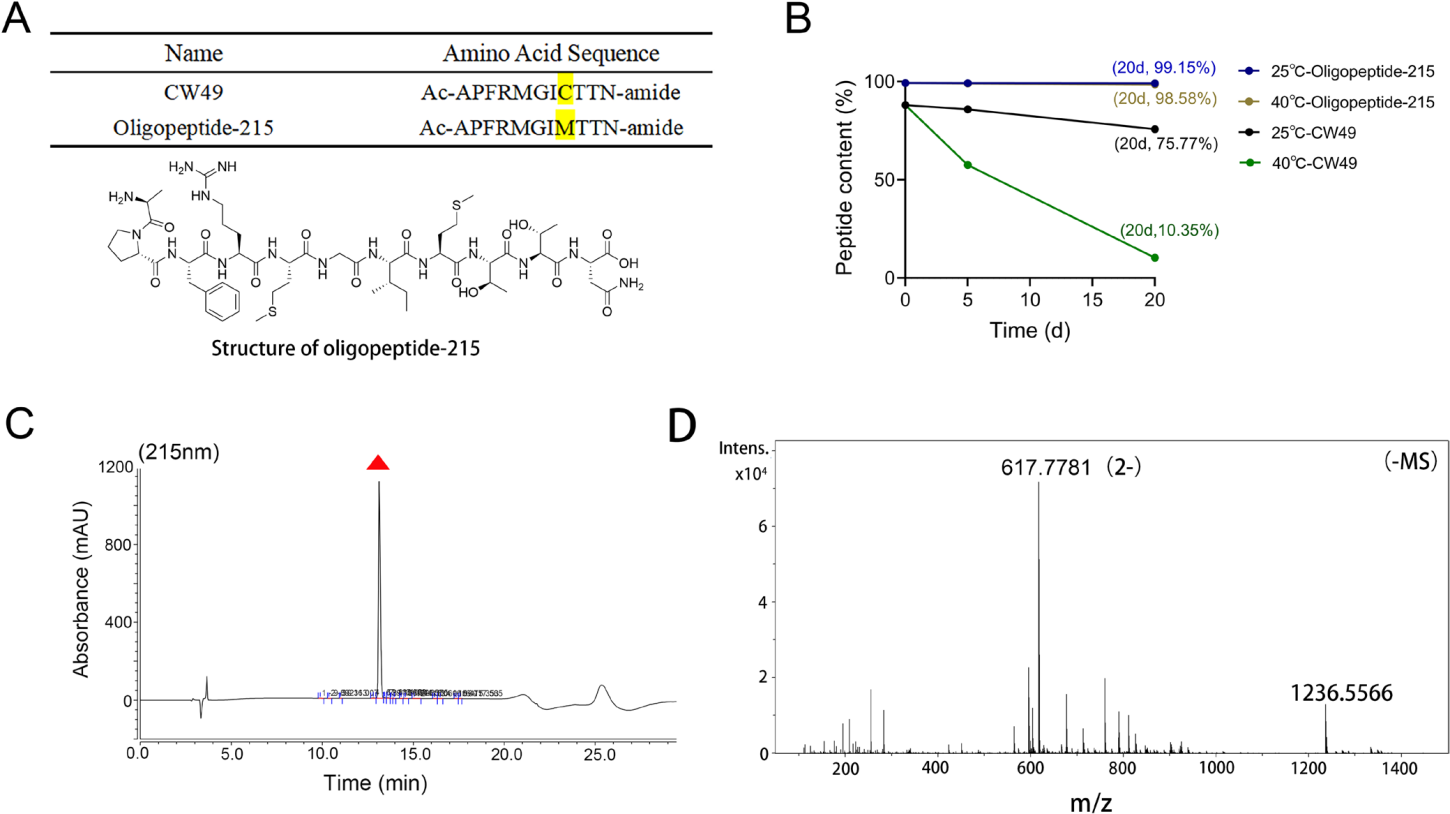
The purification and characterization of Oligopeptide‐215. (A) Amino acid sequences of CW49 and Oligopeptide‐215. (B) The stability tests of CW49 and Oligopeptide‐215 (25°C and 40°C). (C) The purification of Oligopeptide‐215 using RP‐HPLC (column, Vydac, C18, 300 Å, 4.6 × 250 mm), red triangle marked the Oligopeptide‐215. (D) The molecular mass determination of Oligopeptide‐215 by ESI.

Naturally occurring peptides found in animals can serve particular biological activities and play roles as signaling molecules of various physiological processes such as growth, immunity, and migration. Short and easily synthesized peptides with alternative amino acid sequences and combinations have created a new field of molecules inspired by nature and implemented in the cosmetic industry. Recently, we found an oligopeptide, cw49, in the skin secretion of amphibian frogs that has excellent wound‐healing and skin‐repairing activities [26], it contains a cysteine residue in the sequence, which is highly susceptible to oxidation, and almost 90% of the peptide was degraded in 20 days at 40°C during the stability test (Figure 1B). Therefore, we designed to replace cysteine with methionine by amino acid substitution to improve its stability, and the results showed that the new peptide not only could maintain the original biological activity, but also improved the biological stability significantly; both at room temperature of 25°C and at high temperature of 40°C, the new peptide could maintain more than 95% peptide content (Figure 1B), and thus we named the new peptide Oligopeptide‐215 (Figure 1A). Oligopeptide‐215 was purified by using RP‐HPLC (Figure 1C). Accordingly, MS analysis has confirmed the MW of Oligopeptide‐215 as 1237.5566 Da [(617.7781 + 1) × 2 = 1237.556] (Figure 1D), which confirmed that we prepared the target product successfully.

### 3.2 Oligopeptide‐215 Protected and Repaired UVB‐Stimulated HaCaT Cells

**FIGURE 2:**
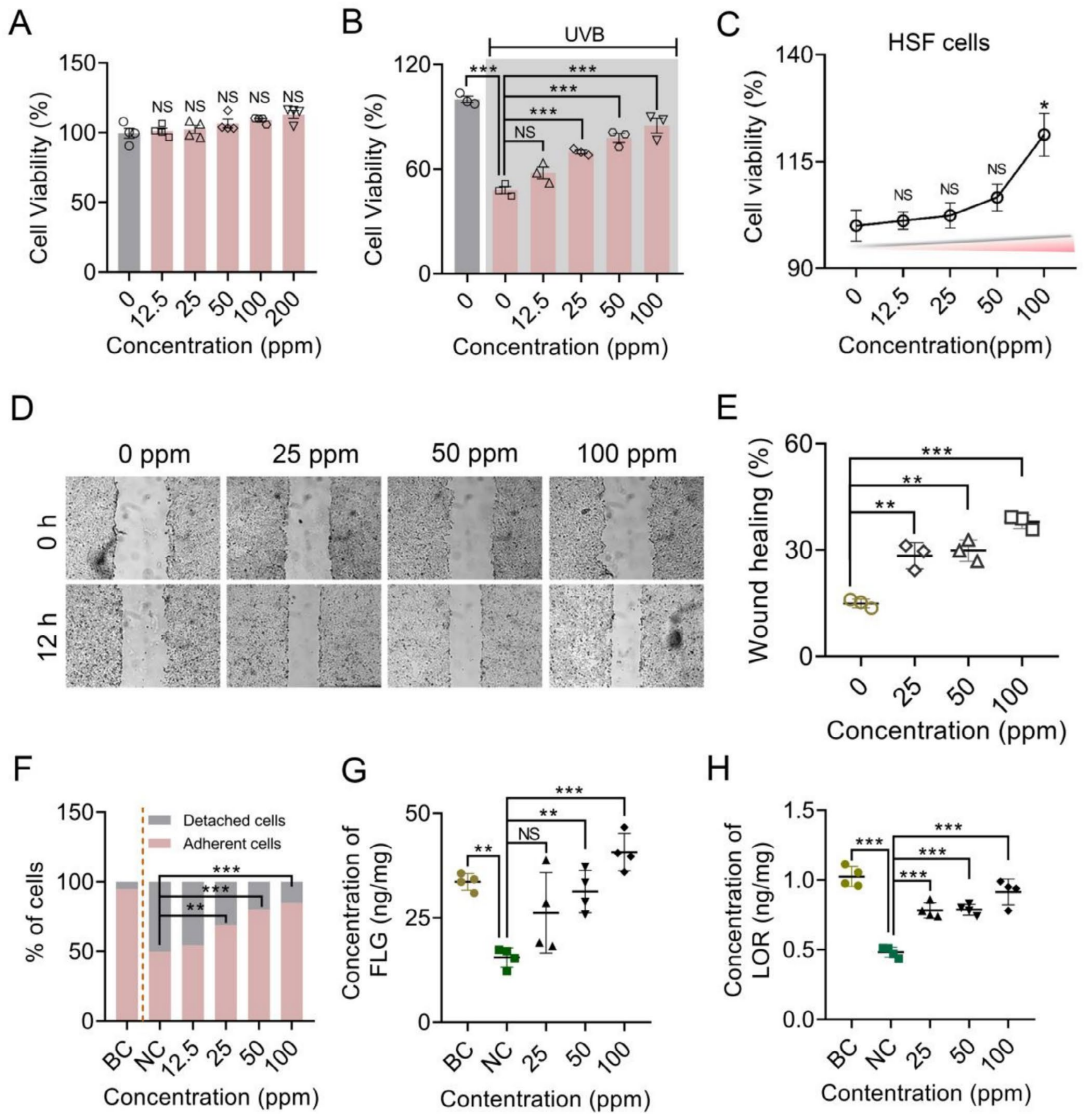
Oligopeptide‐215 repaired HaCaT cells with and without UVB‐irradiation. (A) HaCaT keratinocytes were treated with different doses of Oligopeptide‐215 for 24 h and cytotoxicity was analyzed by MTT assays (*n* = 4). (B) Cells were treated with various doses of Oligopeptide‐215 for 24 h prior to UVB irradiation. Cell viabilities were analyzed using the MTT assays (*n* = 3). (C) HSF cells were subjected to treatment with various concentrations of Oligopeptide‐215 for a duration of 24 h, and their proliferation was subsequently evaluated utilizing MTT assays (*n* = 3). (D) Effect of Oligopeptide‐215 on scratch wound healing with HaCaT cells. Microscopy images of Oligopeptide‐215 increased the wound healing. Confluent cells were treated with various concentrations of Oligopeptide‐215 (25, 50 and 100 ppm). Phase‐contrast micrographs of scratched HaCaT cells were taken at 0 and 12 h after treatment. (E) The wound healing quantitative analysis after scratching for 12 h (*n* = 3). (F) Effect of Oligopeptide‐215 on cell detachment triggered by UVB‐irradiation. Percentage of cell adhesion and detachment relative to control was quantified (*n* = 3). Effect of Oligopeptide‐215 on the expression of FLG (G) and LOR (H) proteins in HaCaT cells under UVB‐damaged (*n* = 4). Data are expressed as the mean ± standard deviation of three independent experiments. NS: Not significant; *p < 0.05; **p < 0.01; ***p < 0.001. BC, blank control; NC, negative control (UVB‐damaged).

To access the cytotoxicity of Oligopeptide‐215, the cell viability assay was performed using MTT test following treatment of HaCaT cells with different doses of Oligopeptide‐215 for 24 h. Oligopeptide‐215 demonstrated no cytotoxicity at concentrations of 12.5–200 ppm (Figure 2A). To investigate the UVB protective effect of Oligopeptide‐215, HaCaT cells were pretreated with Oligopeptide‐215 at different doses prior to UVB (50 mJ/cm^2^) irradiation and cell viability assays were performed. Significant UVB protection was observed in the cells pretreated with 25–100 ppm Oligopeptide‐215 for 24 h (Figure 2B). Although 12.5 ppm of Oligopeptide‐215 pre‐treatment also demonstrated UVB protective capabilities, the improvement in cell viability was not statistically significant compared to the UVB‐irradiated control cells. Also, Oligopeptide‐215 at 100 ppm significantly promoted the proliferation of fibroblast HSF cells (Figure 2C). In order to evaluate the promotive effects of Oligopeptide‐215 on cell adhesion and wound healing, facilitating the evaluation of compounds' efficacy on skin repair, particularly when subjected to UVB irradiation. Given the Oligopeptide‐215's ability to enhance cell viability, we proceeded to investigate its effects on cell adhesion capabilities across various concentrations with UVB irradiation. Upon exposure to UVB light, a decrease in cellular adhesion was observed, accompanied by an increase in detached cells. However, Oligopeptide‐215 treatment significantly mitigated these adverse effects (Figure 2F). To investigate whether Oligopeptide‐215 treatment by regulating cell migration associated with wound healing, scratch assays were conducted in HaCaT cells. Notably, a marked increase in the area of scratch closure was observed in cells treated with Oligopeptide‐215 in comparison to the control group (Figure 2D,E). Exposure of HaCaT cells to UVB radiation resulted in the suppression of FLG and LOR expression, suggesting a disruption in cellular barrier function. As illustrated in Figure 2G,H, the contents of FLG and LOR proteins were notably decreased compared to the corresponding control group following UVB irradiation. However, upon treatment with different concentrations of Oligopeptide‐215, the expression of FLG protein was reinstated (Figure 2G). Similarly, the expression levels of LOR protein also increased significantly in the experimental group treated with Oligopeptide‐215 compared to the model group (Figure 2H). These observations indicated that Oligopeptide‐215 effectively protected and repaired HaCaT cells from UVB‐induced damage by enhancing cell proliferation, migration, adhesion and the expression of skin barrier proteins, thereby preserving the integrity of the cellular barrier.

### 3.3 Oligopeptide‐215 Regulated Skin Barrier Proteins by Inhibiting JAK–STAT Phosphorylation in IL‐4/IL‐13‐Stimulated HaCaT Cells

**FIGURE 3:**
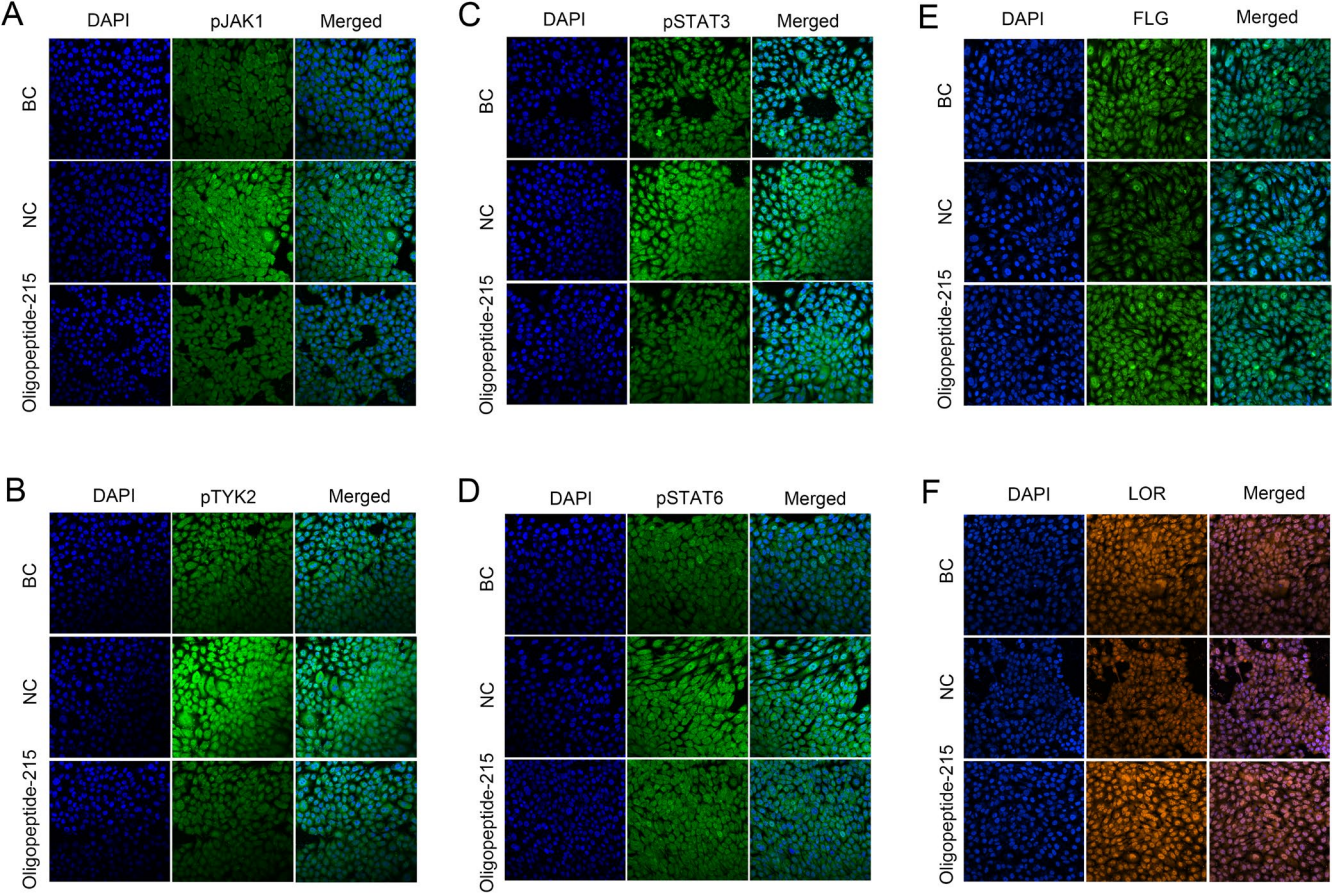
Effects of the Oligopeptide‐215 on IL‐4/IL‐13‐stimulated JAK‐STAT6/STAT3 signaling in HaCaT cells. The HaCaT cells were treated with 100 ppm Oligopeptide‐215 and co‐incubated with IL‐4 (100 ng/mL) and IL‐13 (100 ng/mL) for 24 h. Immunofluorescence staining for p‐JAK1 (A), p‐TYK2 (B), p‐STAT3 (C) and p‐STAT6 (D) expression and localization. DAPI (in blue), p‐JAK1 (in green of A), p‐TYK2 (in green of B), p‐STAT3 (in green of C) and p‐STAT6 (in green of D). Effect of Oligopeptide‐215 on FLG (E) and LOR (F) expression in IL‐4/IL‐13‐stimulated HaCaT cells. DAPI (in blue), FLG (in green of E), LOR (in yellow of F). Signals were visualized and digital images were obtained by using a confocal microscope. BC, blank control; NC, negative control (only IL‐4/IL‐13‐stimulated), Oligopeptide‐215 (100 ppm).

**FIGURE 4:**
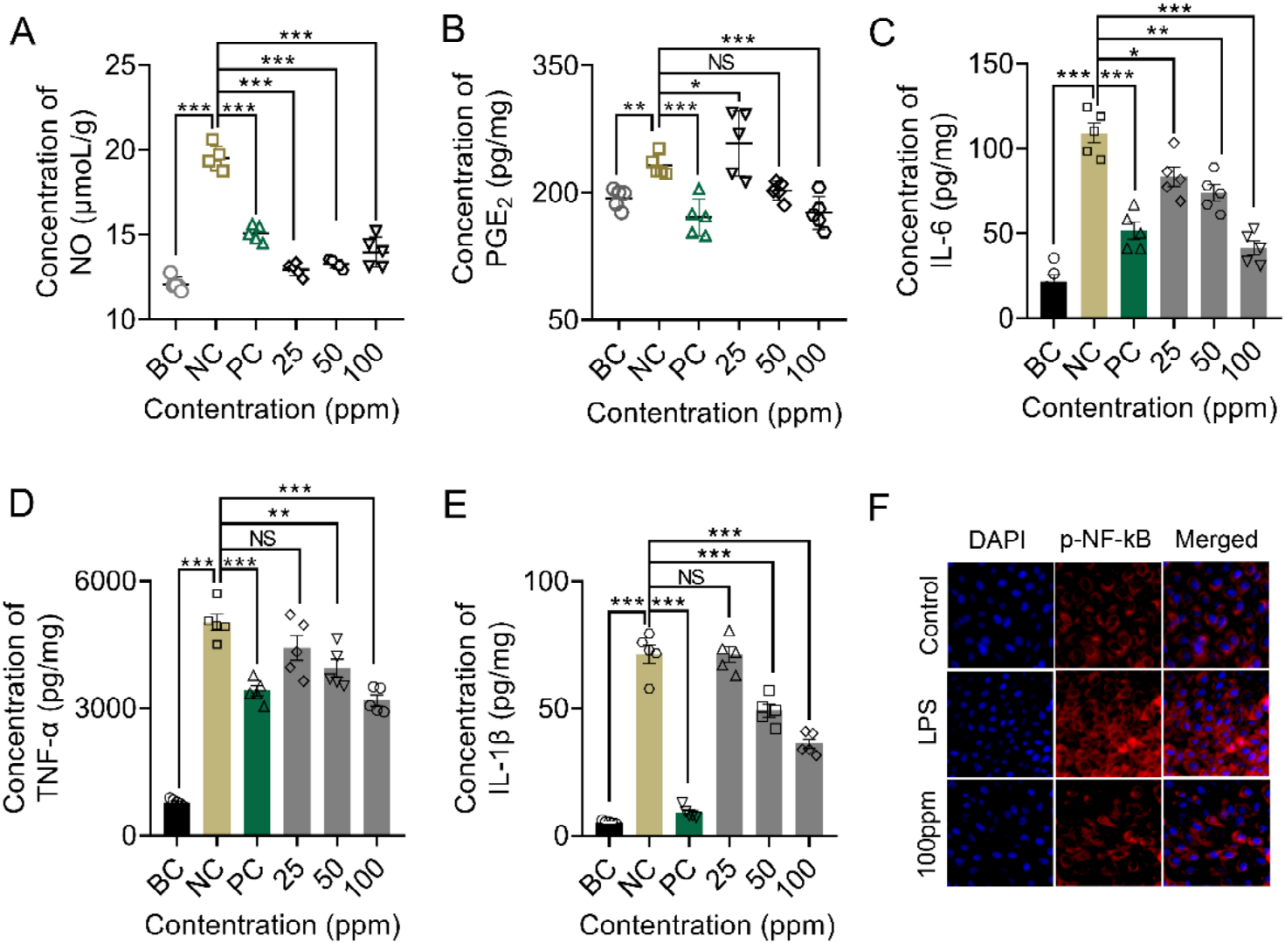
Anti‐inflammatory effects of the Oligopeptide‐215 in LPS‐stimulated RAW264.7 cells. RAW264.7 cells were pretreated with the Oligopeptide‐215 for 48 h, and then exposed to 2 μg/mL LPS for 3 h. (A) NO production. (B) PGE_2_ production. (C–E) IL‐6, TNF‐α, and IL‐1β production. (F) The expression of p‐NF‐kB via immunofluorescence staining. DAPI (in blue) and p‐NF‐kB (in red). Signals were visualized and digital images were obtained by using a confocal microscope. Data are expressed as the mean ± standard deviation of three independent experiments. NS, not significant; *p < 0.05; **p < 0.01; ***p < 0.001. BC, blank control; NC, negative control (2 μg/mL LPS‐treated), PC, positive control (Dexamethasone, DEX).

Under physiological conditions, homeostasis of the skin barrier function is regulated by the coordinated expression of barrier‐related proteins, intercellular lipids, and corneodesmosomes in the granular and cornified layers [27, 28]. In epidermal keratinocytes, IL‐13/IL‐4 bind IL‐4Rα/IL‐13Rα1 heterodimer and activate downstream JAK1/TYK2/JAK2 and then STAT6/STAT3 [29]. In addition, activation of the IL‐13/IL‐4‐JAK‐STAT6/STAT3 pathway inhibits the expression of FLG and LOR [30]. As shown in Figure 2G, we found that Oligopeptide‐215 effectively increased the expression of the skin barrier proteins FLG and LOR. Therefore, we further assessed the expression of p‐JAK and p‐STAT proteins in order to understand the mechanism of the oligopeptide's repairing effect through immunofluorescence. As shown in Figure 3A–D, IL‐4/IL‐13 was able to significantly induce the increased expression of pJAK1, pTYK2, pSTAT6 and pSTAT3 proteins, respectively, when compared to the control group. Administration of Oligopeptide‐215 (100 ppm) significantly decreased the expression of pJAK1, pTYK2, pSTAT6 and pSTAT3 proteins when compared to the negative control group. To investigate the effect of Oligopeptide‐215 on skin barrier function, we treated HaCaT cells with IL‐4/IL‐13 and/or Oligopeptide‐215, and examined whether and how Oligopeptide‐215 modulates skin barrier factors. The results showed that Oligopeptide‐215 restored IL‐4/IL‐13‐suppressed the expression of FLG and LOR (Figure 4E,F). Overall, Oligopeptide‐215 regulated skin barrier function by suppressing the IL‐13/IL‐4‐JAK‐STAT6/STAT3 pathway.

### 3.4 Oligopeptide‐215 Inhibited LPS‐Stimulated Inflammation via NF‐kB Pathway

To investigate the effects of Oligopeptide‐215 on the production of pro‐inflammatory mediators, the RAW264.7 cells were pretreated with the Oligopeptide‐215 for 48 h, and then exposed to 2 μg/mL LPS for 3 h. Oligopeptide‐215 inhibited NO and PGE_2_ (Figure 4A,B) in a dose‐dependent manner. It is well known that NF‐κB is a major pro‐inflammatory regulator. Thus, we further explored whether Oligopeptide‐215 regulates the NF‐κB pathway; we evaluated the expression of p‐NF‐κB protein by immunofluorescence in order to gain insights into the mechanisms of anti‐inflammation effects of Oligopeptide‐215. As shown in Figure 4F, LPS administration was associated with increased expression of p‐NF‐κB protein as compared to the blank control group. Our findings demonstrated that Oligopeptide‐215 effectively attenuated increased expression of p‐NF‐κB. This suggested that Oligopeptide‐215 inhibited the NF‐κB pathway, preventing the subsequent upregulation of inflammatory genes. Furthermore, we examined the impact of Oligopeptide‐215 on the production of downstream inflammatory cytokines. Treatment with Oligopeptide‐215 resulted in a significant suppression of IL‐6, TNF‐α, and IL‐1β (Figure 4C–E) at the protein level. This correlated well with the observed inhibition of NF‐κB activity, further supporting the notion that Oligopeptide‐215 exerts its anti‐inflammatory effects through modulation of the NF‐κB pathway. Taken together, our results suggested that Oligopeptide‐215 exerted anti‐inflammatory effects by regulating the NF‐κB signal pathway.

## 4 Discussion

The epidermal barrier represents a critical interface between organisms and their environment, with its disruption implicated in numerous pathological conditions [31]. Here, we identify Oligopeptide‐215 as a potent regulator of barrier homeostasis, demonstrating its capacity to restore compromised barrier function through coordinated modulation of multiple molecular pathways. Our findings reveal an unexpected mechanism by which this biomimetic peptide regulates both structural and immunological aspects of barrier repair, potentially establishing a new paradigm for barrier protection strategies. Central to our findings is the development of a structurally optimized peptide through strategic amino acid substitution. By replacing the oxidation‐susceptible cysteine residue in the parent peptide CW49 with methionine, we achieved exceptional thermodynamic stability (> 95% retention at 40°C) while preserving biological activity. The remarkable stability profile of Oligopeptide‐215 under physiologically relevant conditions represents a significant advance in biomimetic peptide for barrier protection applications.

Our mechanistic investigations unveiled a sophisticated regulatory network through which Oligopeptide‐215 modulates epidermal barrier function. The Oligopeptide‐215 exhibits precise control over the IL‐13/IL‐4‐JAK‐STAT6/STAT3 signaling axis, a pathway increasingly recognized as central to barrier homeostasis [29]. Recent studies have established that dysregulation of this pathway triggers a cascade of events culminating in compromised barrier integrity [29]. Notably, Oligopeptide‐215 antagonizes IL‐13/IL‐4‐mediated suppression of FLG and LOR expression through concurrent attenuation of JAK1/TYK2 phosphorylation and downstream STAT6/STAT3 activation. This coordinated intervention at multiple signaling nodes represents a significant advancement over existing approaches that typically target individual pathway components. The multi‐target inhibition profile of Oligopeptide‐215 reveals an intriguing mechanism of action. This broad‐spectrum regulation contrasts with conventional small‐molecule JAK inhibitors, which often exhibit selective inhibition of specific JAK family members. Such selective inhibition can trigger compensatory pathway activation, potentially limiting therapeutic efficacy.

Beyond its effects on barrier protein regulation, our investigations reveal that Oligopeptide‐215 exhibits sophisticated control over inflammatory mediators through modulation of the NF‐κB signaling axis. The Oligopeptide‐215 demonstrated remarkable efficacy in attenuating LPS‐induced pro‐inflammatory responses, as evidenced by significant reductions in NO and PGE₂ production. Of particular significance is the peptide's comprehensive regulation of multiple inflammatory cytokines. Oligopeptide‐215 significantly attenuated the expression of TNF‐α, IL‐6, and IL‐1β, key mediators implicated in barrier dysfunction and inflammatory responses. Recent studies have demonstrated that these pro‐inflammatory cytokines can directly compromise barrier integrity through downregulation of tight junction proteins and disruption of lipid metabolism [32]. This anti‐inflammatory capacity is particularly noteworthy given the intricate relationship between barrier dysfunction and inflammatory cascades. Our immunofluorescence analyses revealed that Oligopeptide‐215 effectively suppressed NF‐κB phosphorylation, suggesting direct intervention in this key inflammatory signaling pathway. The ability of Oligopeptide‐215 to simultaneously modulate multiple inflammatory mediators while maintaining barrier protein expression.

These findings collectively establish a molecular framework for understanding barrier regulation and suggest new strategies for intervention. The unique mechanism of action of Oligopeptide‐215, combining structural stability with precise pathway modulation, represents a significant advance in barrier protection approaches. Our work not only provides mechanistic insights into barrier homeostasis but also establishes a foundation for developing more effective strategies for maintaining and restoring barrier integrity.

## 5 Conclusion

Our study reveals an innovative mechanism by which a biomimetic peptide coordinates barrier homeostasis through simultaneous regulation of the physical barrier and the immunologic barrier. The dual capacity of Oligopeptide‐215 to restore barrier protein expression while suppressing inflammatory responses represents a significant advance. The molecular mechanisms uncovered here may have broader implications for understanding barrier regulation in other tissues and could inform the development of targeted therapies for various barrier‐related pathologies. Future studies should explore the clinical treatment of Oligopeptide‐215 and investigate whether similar mechanisms operate in other barrier systems.

## Author Contributions

Conceptualization: Q.Z., C.G., Z.L., and W.D.; software and writing – original draft preparation: Q.Z.; designed the research study: Q.Z., C.G., Z.L., and W.D; contributed essential reagents or tools: W.D.; analyzed the data: Q.Z., C.H., and Y.C. All authors have made substantial, direct, and intellectual contributions to the work, reviewed the manuscript, and approved the final version for publication.

## Funding

The authors have nothing to report.

## Ethics Statement

All procedures involving the research described in this manuscript comply with relevant ethical guidelines and regulations.

## Conflicts of Interest

The authors declare no conflicts of interest.

## Data Availability

Research data are not shared.
