# Correction to “Dual‐Targeting Oligopeptide‐215 Regulates Skin Barrier Homeostasis Through Concurrent Modulation of JAK–STAT and NF‐κB Signaling”

**DOI:** 10.1111/jocd.70976

**Published:** 2026-06-16

Q.Zhang, C.Hu, Y.Chen, et al., “Dual‐Targeting Oligopeptide‐215 Regulates Skin Barrier Homeostasis Through Concurrent Modulation of JAK–STAT and NF‐κB Signaling,” Journal of Cosmetic Dermatology 25, no. 3 (2026): e70704, https://doi.org/10.1111/jocd.70704.

In Figure 1A, the two peptides were incorrectly annotated with N‐terminal acetyl (Ac‐) and C‐terminal amide groups. The correct sequences are unmodified linear peptides and should be annotated as follows:

CW49: H‐APFRMGICTTN‐OH

Oligopeptide‐215: H‐APFRMGIMTTN‐OH

The corrected figure is shown below.

**Figure:**
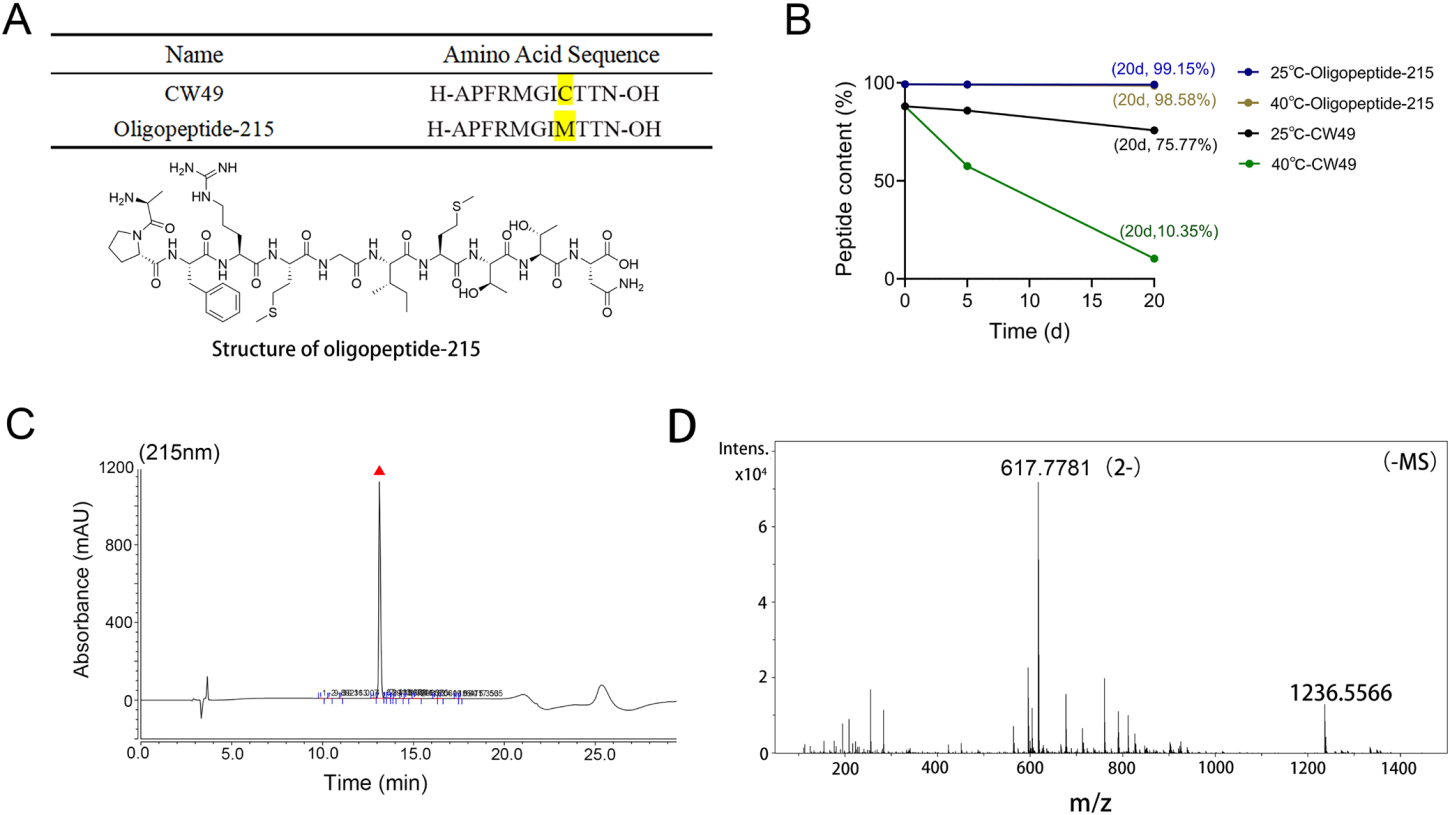
**FIGURE 1** The purification and characterization of Oligopeptide‐215. (A) Amino acid sequences of CW49 and Oligopeptide‐215. (B) The stability tests of CW49 and Oligopeptide‐215 (25°C and 40°C). (C) The purification of Oligopeptide‐215 using RP‐HPLC (column, Vydac, C18, 300 Å, 4.6 × 250 mm); red triangle marked the Oligopeptide‐215. (D) The molecular mass determination of Oligopeptide‐215 by ESI.

We apologize for this error.

